# GHSR gene knockout alleviates the liver pathological response in *Echinococcus granulosus* infection by reducing parasite survival

**DOI:** 10.1186/s13567-025-01478-z

**Published:** 2025-03-11

**Authors:** Jiang Zhu, Tanfang Zhou, Guangfeng Chen, Huijing Gao, Xia Chen, Ayinula Tuohetali, Ya Song, Dongming Pang, Kalibixiati Aimulajiang

**Affiliations:** 1 Department of Abdominal Surgery, The Third People Hospital of Xinjiang Uygur Autonomous Region, Urumqi, 830000 China; 2 State Κey Laboratory of Pathogenesis, Prevention and Treatment of High Incidence Diseases in Central Asia, Clinical Medicine Institute, The First Affiliated Hospital of Xinjiang Medical University, Urumqi, 830054 China; 3 School of Medicine, Shihezi University, Shihezi, 832003 China; 4 College of Veterinary Medicine, Xinjiang Agricultural University, Urumqi, 830052 China

**Keywords:** GHSR, ghrelin, cystic echinococcosis, *Echinococcus granulosus*

## Abstract

Cystic echinococcosis (CE) is a parasitic disease caused by the larval stage of *Echinococcus granulosus*, and the immunosuppressive microenvironment exacerbates disease progression. Ghrelin, a peptide hormone, plays a role in modulating immune inflammation and may influence the progression of *E. granulosus* infection through its receptor, GHSR (growth hormone secretagogue receptor). However, whether GHSR downregulation can inhibit *E. granulosus* infection remains unclear. In this study, we extracted liver tissues from *E. granulosus*-infected mice and those treated with the GHSR antagonist [D-Lys3]-GHRP-6. Proteomic analysis revealed 341 differentially expressed proteins, of which 185 were upregulated and 156 were downregulated. Metabolomic sequencing revealed 101 differentially expressed metabolites, including 62 upregulated and 39 downregulated metabolites. KEGG pathway enrichment analysis of both proteomic and metabolomic data revealed seven key signalling pathways, 11 key proteins, and 26 key metabolites that interact through metabolic and organic system networks. Next, we examined the disease progression of *E. granulosus* infection in GHSR-knockout mice. Compared with the *E. granulosus* (*Eg*) group, the GHSR-KO group presented a significant reduction in the number of liver infection foci. The serum and liver ghrelin levels were significantly greater in the *E. granulosus* group than in the control group, along with increased secretion of proinflammatory cytokines (IL-2 and IFN-γ) and decreased secretion of anti-inflammatory cytokines (IL-4 and IL-10). In contrast, the GHSR-KO group presented significantly lower ghrelin levels in both the serum and liver, with reduced proinflammatory cytokine secretion and increased anti-inflammatory cytokine secretion, similar to those of the control group. Furthermore, ghrelin and inflammation-related factors, including MyD88, NF-κB p65, iNOS, and Arg-1, exhibited coordinated expression changes in liver lesions and surrounding areas. These findings suggest that GHSR gene knockout can ameliorate the progression of liver *E. granulosus* infection and associated liver inflammation.

## Introduction

Cystic echinococcosis (CE) is a zoonotic liver disease caused by *E. granulosus* infection [1]. Over the past two decades, CE has become a significant global health concern, with high incidence rates reported in regions such as Western China, Central Asia, South America, the Mediterranean, and East Africa. The primary risk factors for CE include exposure to dogs and livestock farming [2, 3]. CE typically causes irreversible damage to the liver, including inflammation, tissue injury, and fibrosis. The activation of the MyD88/NF-κB signalling pathway, along with T-cell-driven proinflammatory responses, plays a critical role in activating the host’s innate immunity to combat echinococcosis [4]. However, when immune inflammation is suppressed, the disease progresses rapidly [5]. At present, CE diagnostic procedures have limitations, drug toxicity and side effects, surgical strategies are often insufficient, and control and prevention measures are also facing challenges [1]. Consequently, further research into the pathogenesis of CE is crucial for identifying novel therapeutic targets to combat this disease.

Ghrelin, an endogenous ligand for the growth hormone secretagogue receptor (GHSR), is primarily secreted by the gastric fundus, although various liver cells also secrete it. Ghrelin plays a pivotal role in regulating growth, metabolism, and glucose‒lipid homeostasis while exerting significant effects on liver immune inflammation and fibrosis [6–8]. Ghrelin requires binding to its receptor, GHSR, to exert its biological effects, and its ligand, GHRP, can activate GHSR [9]. Studies have shown that ghrelin can inhibit the MyD88/NF-κB inflammatory signalling pathway [10–12] and the TGF-β/Smad3 fibrosis signalling pathway [13, 14], offering protective effects in diseases such as non-alcoholic fatty liver disease, hepatitis, liver fibrosis, cirrhosis, and liver injury by improving immune inflammation and fibrosis. However, our study revealed that during the early stages of *E. granulosus* liver infection, ghrelin expression increases, potentially modulating the inflammatory microenvironment and facilitating immune evasion by the parasite, thus promoting the progression of the infection [7]. Blocking the ghrelin receptor GHSR has been shown to inhibit the growth of *E. granulosus* in vitro [10, 15], suggesting that inhibiting GHSR expression may affect the progression of *E. granulosus* liver infection. However, whether GHSR inhibition can reduce its expression and inhibit the infection process remains unexplored, necessitating further in vivo studies to determine its precise role and potential therapeutic effects in the infection process.

Proteomic research on CE has revealed new protein functions that contribute to a better understanding of cellular processes, signalling pathways, and metabolic networks, thereby elucidating the molecular mechanisms of this disease and enhancing the understanding of its biochemical and immunological characteristics [16–18]. The proteomic profile of *E. granulosus* and its expression pattern, along with host‒parasite interactions, are essential for understanding the biology of the parasite, ultimately assisting in drug design and vaccine development [19]. Metabolomic studies are similarly valuable for identifying biomarkers associated with CE and determining the key amino acid and lipid metabolic pathways involved in disease pathogenesis, thereby enhancing the understanding of patient behavior in clinical settings [20]. Combined proteomic and metabolomic analysis offers more comprehensive insights into the key molecular mechanisms underlying *E. granulosus* infection [21, 22].

In this study, we employed proteomic and metabolomic sequencing to analyse differentially expressed proteins and metabolites in the livers of *E. granulosus*-infected mice and those treated with [D-Lys3]-GHRP-6, a GHSR antagonist. We aimed to investigate whether genetic knockout of the ghrelin receptor GHSR could improve disease progression in liver *E. granulosus* infection and mitigate liver inflammation induced by infection. The findings of this study may contribute to the identification of new drug targets for immune-based therapies in CE by exploring growth and metabolic pathways.

## Materials and methods

### Animals

In this study, 6- to 8-week-old female C57BL/6 mice weighing 20 ± 2 g were provided by Shanghai Southern Model Biology Technology Co., Ltd. All the mice were housed in a specific pathogen-free (SPF) barrier environment at the Animal Experiment Center of Xinjiang Medical University. The plants were maintained under controlled conditions of constant temperature and humidity with a 12-h light/dark cycle and unrestricted access to food and water. Prior to the experiment, the mice were fasted overnight and deprived of water. The animal experimental protocol was reviewed and approved by the Animal Ethics Committee of the First Affiliated Hospital of Xinjiang Medical University (Ethics Approval No: IACUC-20230321014).

### Isolation and culture of protoscoleces (PSCs)

Infected *Echinococcus granulosus* liver tissues were obtained from a local slaughterhouse in sheep following standard disinfection procedures. The liver tissues were wiped with dry paper towels before the cyst fluid and vesicular tissue were collected under sterile conditions. Protoscoleces (PSCs) were isolated by grinding, washing, and filtering. The PSCs were digested with 1% pepsin in physiological saline at 37 °C for 30 min, and the pH was adjusted to 3.0 [23]. Cell viability was assessed by eosin staining, with only PSCs demonstrating > 95% viability being used for subsequent in vivo experiments.

### Mouse model of *E. granulosus* infection

The mice were anaesthetized via isoflurane inhalation before being subjected to laparotomy. C57BL/6 mice were initially divided into two groups (*n* = 6) on the basis of the type of intervention: the Model group and the GHRP-6 group. In the model group, 2000 PSCs were injected into the hepatic portal vein. In the GHRP-6 group, 2000 PSCs were injected into the hepatic portal vein in the same way, after which the ghrelin receptor antagonist [D-Lys3]-GHRP-6 (Abcam, Cambridge, MA, USA) was administered by intraperitoneal injection. The treatment lasted for 90 days, with a daily dose of 100 μg per mouse [24]. After the intervention period, the mice were euthanized via isoflurane inhalation followed by cervical dislocation, and liver samples were collected.

The mice were subsequently divided into three groups (*n* = 6) on the basis of the intervention: the control group (healthy C57BL/6 mice), the *E. granulosus* group (C57BL/6 mice infected with *E. granulosus* in the liver), and the *E. granulosus* (GHSR^−/−^) group (GHSR knockout C57BL/6 mice infected with *E. granulosus*). In the control group, an equal volume of physiological saline was injected into the portal vein, whereas in the *E. granulosus* and *E. granulosus* (GHSR^−/−^) groups, 0.1 mL of physiological saline containing 4000 PSCs was injected into the portal vein [23, 25, 26].

The experiment lasted for 90 days, with euthanasia performed at the end of the experiment. The data collected included body weight, liver weight, spleen weight, and the number of cystic lesions on the liver surface. Serum and liver samples were also collected at euthanasia.

### Enzyme-linked immunosorbent assay (ELISA)

The levels of IL-2, IFN-γ, IL-4, and IL-10 in liver tissues were measured using commercially available ELISA kits following the manufacturer’s instructions. The absorbance was read at 450 nm using a BioTek microplate reader (BioTek, Vermont, USA). All kits were obtained from Lapuda Biotechnology. (Nanjing, China).

### Immunohistochemical (IHC) and haematoxylin and eosin (H&E) staining

Mouse liver samples were fixed in 4% formalin (Biosharp, Hefei, China) to preserve tissue integrity. Following fixation, the liver tissues were processed, embedded in paraffin, and sectioned for subsequent staining. H&E staining was performed to evaluate general tissue morphology, while IHC staining was conducted to assess protein expression.

For IHC, the slides were deparaffinized and rehydrated using a gradient alcohol series, followed by antigen retrieval with Tris–EDTA and citrate buffer according to the manufacturer’s protocol. Endogenous peroxidase activity was blocked with 3% hydrogen peroxide, and the slides were incubated with goat serum for 30 min (Proteintech, Wuhan, China). Primary antibodies were applied overnight at 4 °C, followed by incubation with a goat anti-rabbit/mouse HRP-labelled polymer (Proteintech, Wuhan, China). Color development was achieved using a DAB kit (Proteintech, Wuhan, China), and the slides were counterstained with hematoxylin (ZSGB-Bio, Beijing, China). Microscopic examination was conducted using an optical microscope (Olympus, Tokyo, Japan), and images were captured. The positively stained areas were quantified using Image-Pro Plus 6.0 software (Media Cybernetics, USA). The primary antibodies used included anti-rabbit ghrelin (1:7000, ab20979), anti-rabbit NF-κB p65 (1:1000, ab16502), and anti-rabbit iNOS (1:100, ab115819) (Proteintech, Wuhan, China). Anti-rabbit Arg-1 (1:200, 16001-1-AP) and anti-rabbit MyD88 (1:200, 67969-1-Ig) (Abcam, Cambridge, MA, USA) were used.

### Proteomic analysis

Liver samples from the Model and GHRP groups were collected, and three mice from each group were randomly selected for proteomic analysis. The liver tissues were homogenized in liquid nitrogen and then treated with 1 mL of precooled propanol. Ultrasonic oscillation was performed for 15 min, followed by centrifugation at 21 300 × *g* for 30 min at 4 °C. The supernatant was discarded, and the precipitate was washed with acetone before being centrifuged at 14 000 × *g* for 15 min. After acetone evaporation, the samples were dissolved in precooled lysis buffer. The protein concentration was determined using the BCA assay. SDS‒PAGE was performed to assess protein extraction efficiency. Enzymatic digestion and desalting were performed using the MagicOmics Micro Proteomics Sample Preparation Kit (QLBio, Beijing, China). Proteins were digested and analysed by liquid chromatography‒tandem mass spectrometry (LC‒MS/MS) on an Orbitrap Exploris™ 480 mass spectrometer (Thermo Fisher Scientific, MA, USA). Differentially expressed proteins were functionally annotated using Gene Ontology (GO) enrichment analysis and Kyoto Encyclopedia of Genes and Genomes (KEGG) pathway analysis. Proteins with log_2_-fold changes ≥ 1 or ≤  − 1 and a false discovery rate (FDR) < 0.05 were considered significantly up- or downregulated [27, 28].

### Metabolomics analysis

Metabolomic analysis was conducted alongside proteomic sequencing of liver tissues from the Model and GHRP groups. Liver tissue (100 mg) was homogenized in a mixture of 75% methanol: 25% H_2_O with steel beads for 30 min at room temperature. The mixture was then centrifuged, and the supernatant was collected and concentrated. The samples were redissolved in 50% acetonitrile solution and analysed by LC‒MS via a Vanquish liquid chromatography system (Thermo Fisher Scientific, MA, USA) and an Orbitrap Exploris 120 mass spectrometer (Thermo Fisher Scientific, MA, USA). The resulting data were compared with metabolite databases (HMDB, MassBank, and KEGG) for metabolite identification. Differentially abundant metabolites were identified on the basis of |log_2_FoldChange|≥ 1 and *P* < 0.05 [29].

### Combined proteomic and metabolomic analysis

A combined analysis of proteomics and metabolomics was performed for the Model and GHRP groups. Differential proteins and metabolites were annotated in the KEGG database, and two-way orthogonal partial least squares (O2PLS) analysis was conducted to evaluate sample quality and histological relevance. KEGG enrichment analysis was subsequently performed to identify significantly enriched pathways. Integration of proteomic and metabolomic clustering analysis was used to investigate regulatory mechanisms between genes and metabolites.

### Statistical analysis

Statistical analysis was performed using SPSS v26.0 software (IBM, USA), and graphical representations were generated using GraphPad Prism 9.0 software (GraphPad Software, USA). The data are presented as mean ± standard deviation, with categorical variables expressed as frequencies or percentages. Statistical significance between groups was assessed using t tests or one-way analysis of variance (ANOVA) on the basis of results from at least three independent experiments. Spearman’s correlation coefficient was used for correlation analysis. A *P* value of < 0.05 was considered statistically significant.

## Results

### Differential expression analysis of the GHSR ligand GHRP and model proteomics

**Figure 1:**
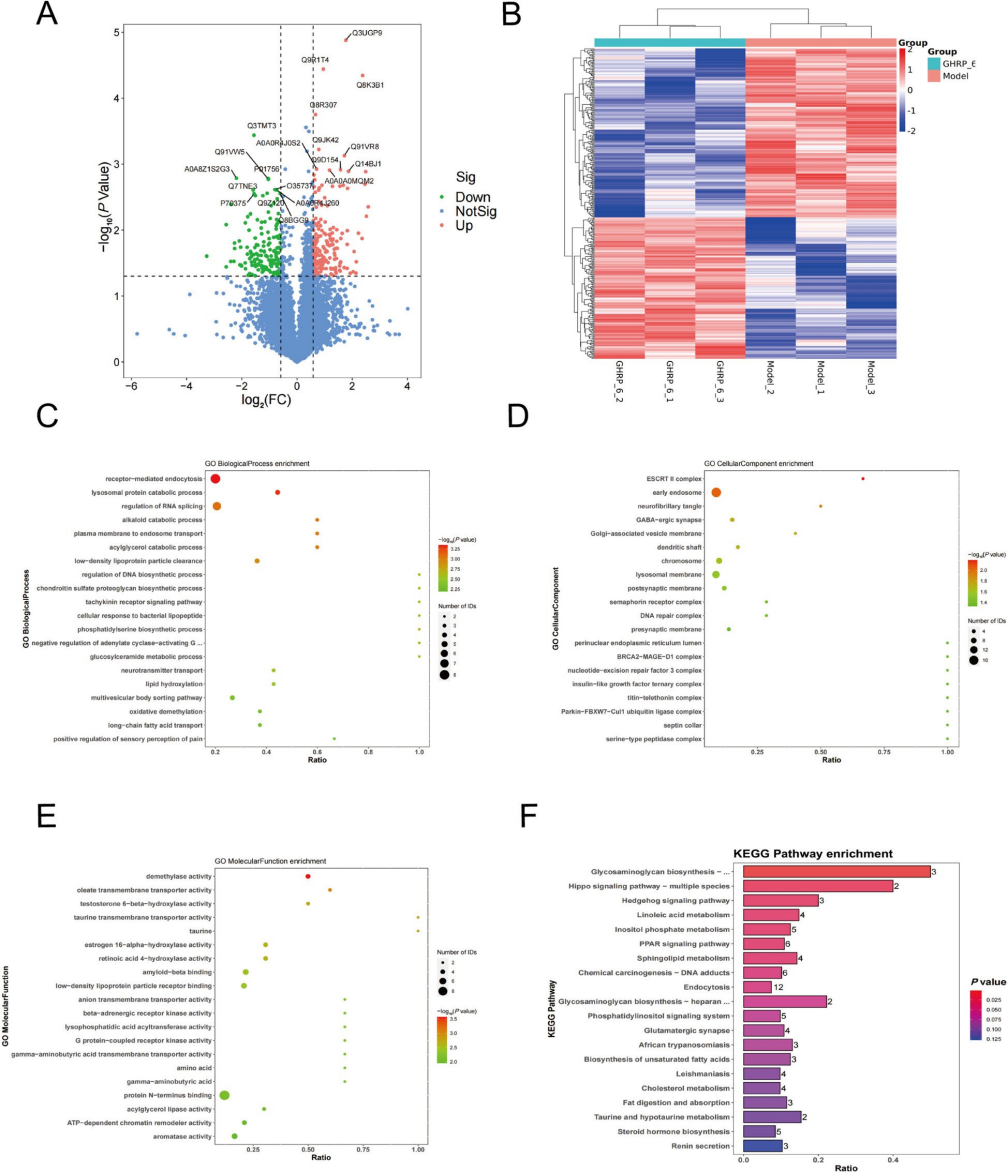
**Differential expression analysis of the GHSR ligand GHRP and model proteomics. A** Volcano plot of differential proteins, where red dots indicate upregulated metabolites, blue dots indicate downregulated metabolites, and blue dots represent metabolites that do not meet the differential screening criteria. **B** Heatmap of differential proteins, with color indicating correlation; red denotes positive correlation, and blue denotes negative correlation, with deeper colors signifying stronger correlations. **C** Bubble chart displaying enrichment analysis of biological processes for differential proteins. The size of the bubbles represents the number of metabolites associated with the pathway, and the colour indicates the *p* value, with red signifying a smaller *p* value and green indicating a larger *p* value. **D** Bubble chart showing enrichment analysis of cellular components for differential proteins. **E** Bubble chart displaying the enrichment analysis of the molecular functions of the differentially expressed proteins. (F) Histogram illustrating the results of the KEGG pathway enrichment analysis of the differentially expressed proteins. Data were considered statistically significant: *P < 0.05; **P < 0.01; ***P < 0.001; ****P < 0.0001.

Quantitative analysis of liver tissues from the Model and GHRP groups was performed using LC–MS/MS label-free quantitative proteomics, resulting in the identification of 341 differentially expressed proteins. Among these proteins, 185 were significantly upregulated, whereas 156 were downregulated (Figures 1A, B). The top 10 differentially expressed proteins, ranked by fold change (FC), are presented in Table 1. Biological process analysis revealed significant enrichment in processes such as biosynthesis, gene expression, RNA processing, cell cycle regulation, and metabolism (Figure 1C). Gene Ontology (GO) enrichment analysis indicated that these differentially expressed proteins were predominantly localized in the cell membrane and organelles (Figure 1D). Furthermore, molecular function analysis highlighted activities such as aromatase activity, acylglycerol lipase activity, demethylase activity, and receptor kinase activity (Figure 1E). KEGG pathway analysis revealed significant enrichment of these proteins in pathways related to PPAR signalling, mineral homeostasis, endocytosis, and bile secretion (Figure 1F).

**Table 1:** **The differential upregulated and downregulated proteins**

Protein name	Expression type	FC	*P* value
D3YZ06	Up	5.97693	0.00444
P26350	Up	5.71370	0.00617
A0A0U1RNW0	Up	5.60635	0.00130
Q8VEB1	Up	5.50443	0.00202
Q8K3B1	Up	5.19093	0.00005
H3BKL1	Up	5.11648	0.01340
O35710	Up	4.68260	0.01114
A0A0J9YTR2	Up	4.43679	0.03029
B2RTJ2	Up	4.40167	0.04495
D3Z6P0	Up	4.18724	0.02112
Q5F2E7	Down	0.23472	0.01460
A0A8Z1S2G3	Down	0.21804	0.00163
Q9JHS9	Down	0.20882	0.01899
F6UFZ5	Down	0.20825	0.01571
B2RVZ0	Down	0.20489	0.02983
A0A140LIS7	Down	0.18960	0.00406
A0A1Y7VKT9	Down	0.18941	0.02991
A0A075B5Y1	Down	0.16892	0.03613
P56393	Down	0.16881	0.00828
Q9JJU9	Down	0.10360	0.02481

### Differential analysis of the GHSR ligand GHRP and model metabolites

**Figure 2:**
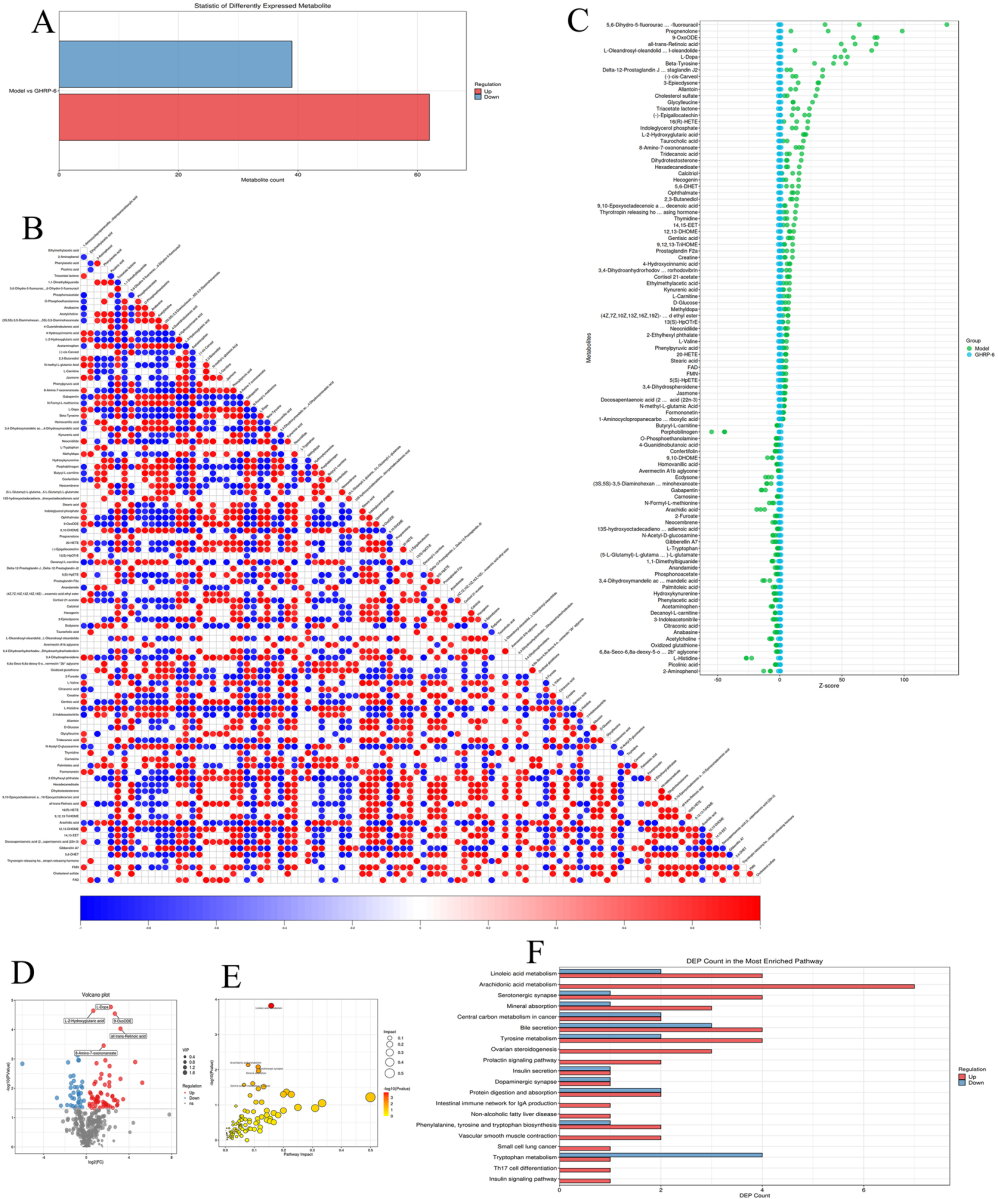
**Differential analysis of the GHSR ligand GHRP and model metabolites. A** Metabolite differential statistics plot, where red indicates the upregulation of metabolites and green indicates the downregulation of metabolites. **B** Hierarchical clustering heatmap of differentially abundant metabolites, with red representing high expression and blue representing low expression. **C** Z score bubble plot of differentially abundant metabolites, utilizing the Z score as the horizontal axis and the negative logarithm of the adjusted *p* value as the vertical axis (y-axis), with higher values indicating more significant differences. **D** Volcano plot of differentially abundant metabolites, with red dots indicating upregulated metabolites, blue dots indicating downregulated metabolites, and gray dots representing metabolites that do not meet the differential screening criteria. **E** Bubble chart displaying the results of the KEGG pathway enrichment analysis of the differentially abundant metabolites. The size of the bubbles represents the number of metabolites associated with the pathway, and the colour indicates the *p* value. Redder colours signify lower *p* values, whereas bluer colours indicate higher *p* values. **F** Histogram showing the count of KEGG pathway differential molecules, with red representing upregulated metabolites and green representing downregulated metabolites. Data were considered statistically significant: *P < 0.05; **P < 0.01; ***P < 0.001; ****P < 0.0001.

Metabolomic analysis of liver tissues from the Model and GHRP groups was conducted by LC‒MS/MS label-free quantitative metabolomics. A total of 440 metabolites were identified, with 101 showing differential expression, including 62 upregulated and 39 downregulated metabolites (Figures 2A, D). The heatmap of differentially abundant metabolite correlations further supported these findings, with clustering trees displayed on both axes, where red denotes a strong correlation and blue represents a weak correlation (Figure 2B). The Z score bubble plot for differentially abundant metabolites revealed that, compared with the Model group, the GHRP group presented higher Z scores for fluorodeoxyuridine and 9-OXOODE. Pearson correlation coefficients were also calculated to assess the relationships between individual metabolites in both groups (Figure 2C). KEGG pathway enrichment analysis highlighted key metabolic pathways with significant differences between the two groups, including arachidonic acid metabolism, serotonergic synapses, and linoleic acid metabolism (Figure 2E). Extreme significantly enriched metabolic pathways are presented in Table 2. Molecular analysis of these pathways revealed a greater number of upregulated molecules in the arachidonic acid metabolism pathway than in the tryptophan metabolism pathway (Figure 2F).

**Table 2:** **Extreme significantly differential enriched metabolic pathways**

Differential pathways	Hit	*P* value	FDR
Arachidonic acid metabolism	7/25	0.00718	0.72984
Bile secretion	7/97	0.02700	1.00000
Linoleic acid metabolism	6/28	0.00015	0.04098
Tyrosine metabolism	6/78	0.03016	1.00000
Serotonergic synapse	5/42	0.00826	0.72984
Tryptophan metabolism	5/83	0.10700	1.00000
Mineral absorption	4/29	0.01073	0.72984
Central carbon metabolism in cancer	4/37	0.02480	1.00000
Protein digestion and absorption	4/47	0.05325	1.00000
ABC transporters	4/138	0.61358	1.00000
Ovarian steroidogenesis	3/24	0.03493	1.00000
Aminoacyl-tRNA biosynthesis	3/52	0.20998	1.00000
Neuroactive ligand‒receptor interaction	3/52	0.20998	1.00000
Cysteine and methionine metabolism	3/63	0.30087	1.00000
Biosynthesis of unsaturated fatty acids	3/74	0.39361	1.00000
Steroid hormone biosynthesis	3/99	0.58734	1.00000
Prolactin signalling pathway	2/11	0.04226	1.00000
Insulin secretion	2/12	0.04972	1.00000
Dopaminergic synapse	2/12	0.04972	1.00000
Riboflavin metabolism	2/20	0.12250	0.91185

### Analysis combining proteomics and metabolomics

**Figure 3:**
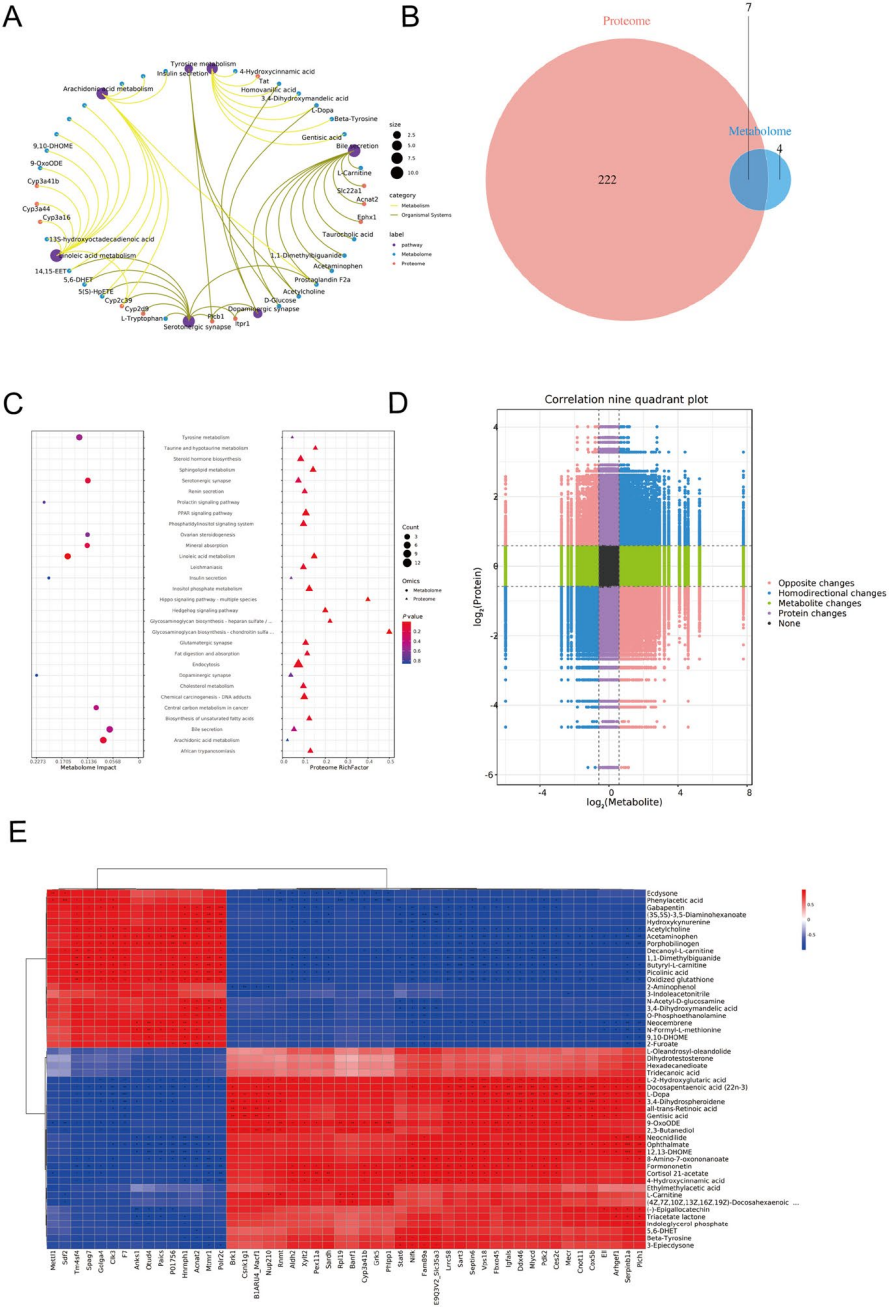
**Analysis combining proteomics and metabolomics. A** KEGG network interaction plot. The purple dots represent signalling pathways, the blue dots represent metabolomics, and the orange dots represent proteomics. Lines connecting two proteins indicate interactions, with line colors reflecting the type of interaction, where yellow lines denote metabolism and green lines denote organ systems. **B** KEGG Venn diagram, with orange circles representing the proteome and blue circles representing the metabolome, with the intersection of the two circles representing the significant differential genes common to both groups. **C** KEGG enrichment bubble chart displaying the magnitude of *p* values, where a redder color indicates a stronger level of enrichment. The size of the dots represents the number of differential proteins or metabolites. The circle on the left represents the metabolome, whereas the triangle on the right represents the proteome. If a pathway corresponds to both a triangle and a circle, the pathway is enriched in both histologies. **D** Correlation nine-quadrant plot showing different trends in metabolite and protein changes: orange dots indicate opposite trends, blue dots indicate consistent trends, green dots indicate metabolic differences, purple dots indicate protein differences, and black dots indicate no differences in either metabolism or protein. **E** Correlation heatmap, where a redder colour indicates a stronger positive correlation and a bluer colour indicates a stronger negative correlation. Data were considered statistically significant: *P < 0.05; **P < 0.01; ***P < 0.001; ****P < 0.0001.

To further elucidate the roles of differential proteins and metabolites in cystic echinococcosis, proteomics and metabolomics data were integrated. KEGG network interaction maps were used to identify key molecules and pathways critical for understanding biological processes and disease mechanisms. This analysis revealed seven major signalling pathways, 11 key proteins, and 26 essential metabolites that interact through metabolic processes and organ systems (Figure 3A). A Venn diagram was used to visualize the overlap between differentially expressed proteins and metabolites, highlighting 222 proteins unique to proteomics, 4 unique to metabolomics, and 7 shared between both datasets (Figure 3B). A bubble chart visualizing enriched pathways in both datasets revealed 26 pathways enriched in proteomics and 11 enriched in metabolomics, with seven common pathways shared between the two (Figure 3C). A nine-quadrant plot showing the distribution of significance levels across different subsets further emphasized interactions between differential proteins and metabolites (Figure 3D). Additionally, clustering analysis of the top 50 proteins and metabolites with the lowest *p* values resulted in a correlation heatmap (Figure 3E), providing a comprehensive view of the molecular interactions and pathways involved in the pathogenesis of cystic echinococcosis.

### GHSR deficiency improves the progression of hepatic *E. granulosus* infection

**Figure 4:**
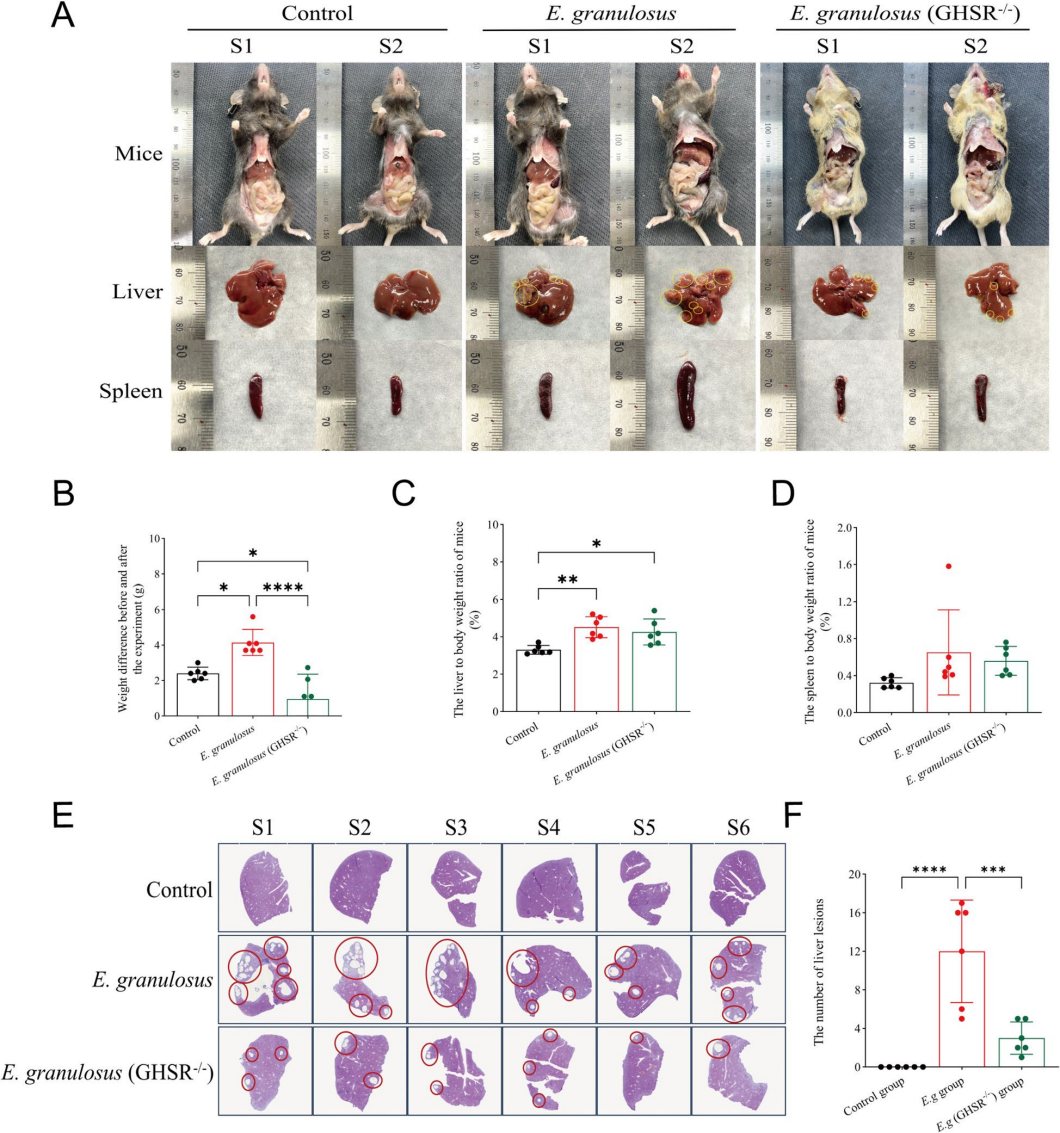
**GHSR deficiency improves the progression of hepatic *E. granulosus* infection. A** Schematic diagram of the mouse liver and spleen. The red circle represents the location of the infected lesion on the surface of the liver. **B** Intergroup analysis of the body weights of the mice before and after the experiment. **C** Intergroup analysis of the liver-to-body weight ratio at the time of mouse euthanasia. **D** Intergroup analysis of the spleen-to-body weight ratio at the time of mouse euthanasia. **E** H&E staining images of liver sections. **F** Intergroup analysis of the number of liver lesions observed by H&E staining of liver sections. Data were considered statistically significant: *P < 0.05; **P < 0.01; ***P < 0.001; ****P < 0.0001.

Our previous studies indicated that blocking ghrelin receptor activity may promote the death of *E. granulosus* larvae, potentially slowing the progression of liver infection [15]. To confirm this, we used GHSR knockout (GHSR^−/−^) mice to simulate liver infection by *E. granulosus*. After 90 days, the number of cystic lesions on the liver surface was significantly lower in the GHSR^−/−^ group than in the *E. granulosus* group (Figure 4A). Additionally, the GHSR^−/−^ group presented a significant reduction in spleen volume and a smaller weight change throughout the experiment, which is consistent with the known effects of ghrelin receptor blockade on weight loss (Figure 4B). Although the liver-to-body weight ratio in the GHSR^−/−^ group was lower than that in the control group, no significant difference was observed (Figure 4C). Similar trends were observed for the spleen-to-body weight ratio (Figure 4D). Histological examination of the left liver lobe via H&E staining revealed pronounced infection lesions in the *E. granulosus* group. Notably, the total number of liver lesions (including cystic and fibrotic lesions) in the GHSR^−/−^ group was significantly lower than that in the *E. granulosus* group (Figures 4E, F), supporting the hypothesis that blocking GHSR receptor activity can mitigate the progression of liver infection by *E. granulosus*.

### Deletion of the GHSR gene reduces ghrelin levels in the serum and liver of *E. granulosus*-infected individuals

**Figure 5:**
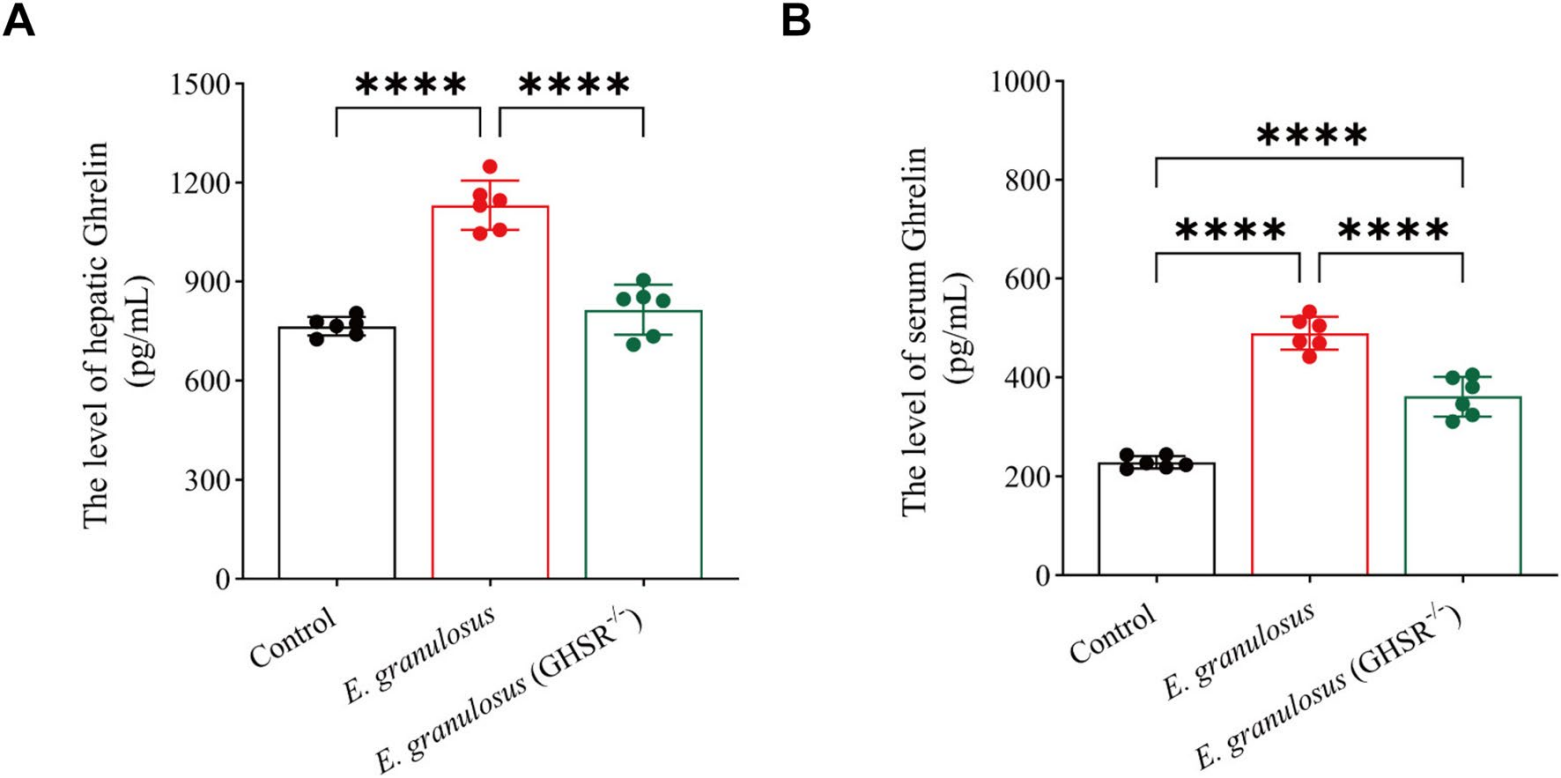
**Deletion of the GHSR gene reduces ghrelin levels in the serum and liver of* E. granulosus*-infected individuals. A** ELISA analysis of serum ghrelin levels and comparisons between groups. **B** ELISA analysis of liver ghrelin levels and comparisons between groups. Data were considered statistically significant: *P < 0.05; **P < 0.01; ***P < 0.001; ****P < 0.0001.

The impact of GHSR knockout on ghrelin levels in the serum and liver was assessed via ELISA. The results revealed that serum ghrelin levels were significantly elevated in the *E. granulosus*-infected mice, with GHSR^−/−^ mice showing notably higher ghrelin levels than the control group did (Figure 5A). Similarly, ghrelin levels in the liver were significantly elevated in the *E. granulosus* group (Figure 5B). These findings suggest that the absence of GHSR leads to altered ghrelin regulation, which may contribute to the observed differences in infection progression and liver pathology.

### GHSR deficiency alleviates the hepatic inflammatory response in *E. granulosus* infection

**Figure 6:**
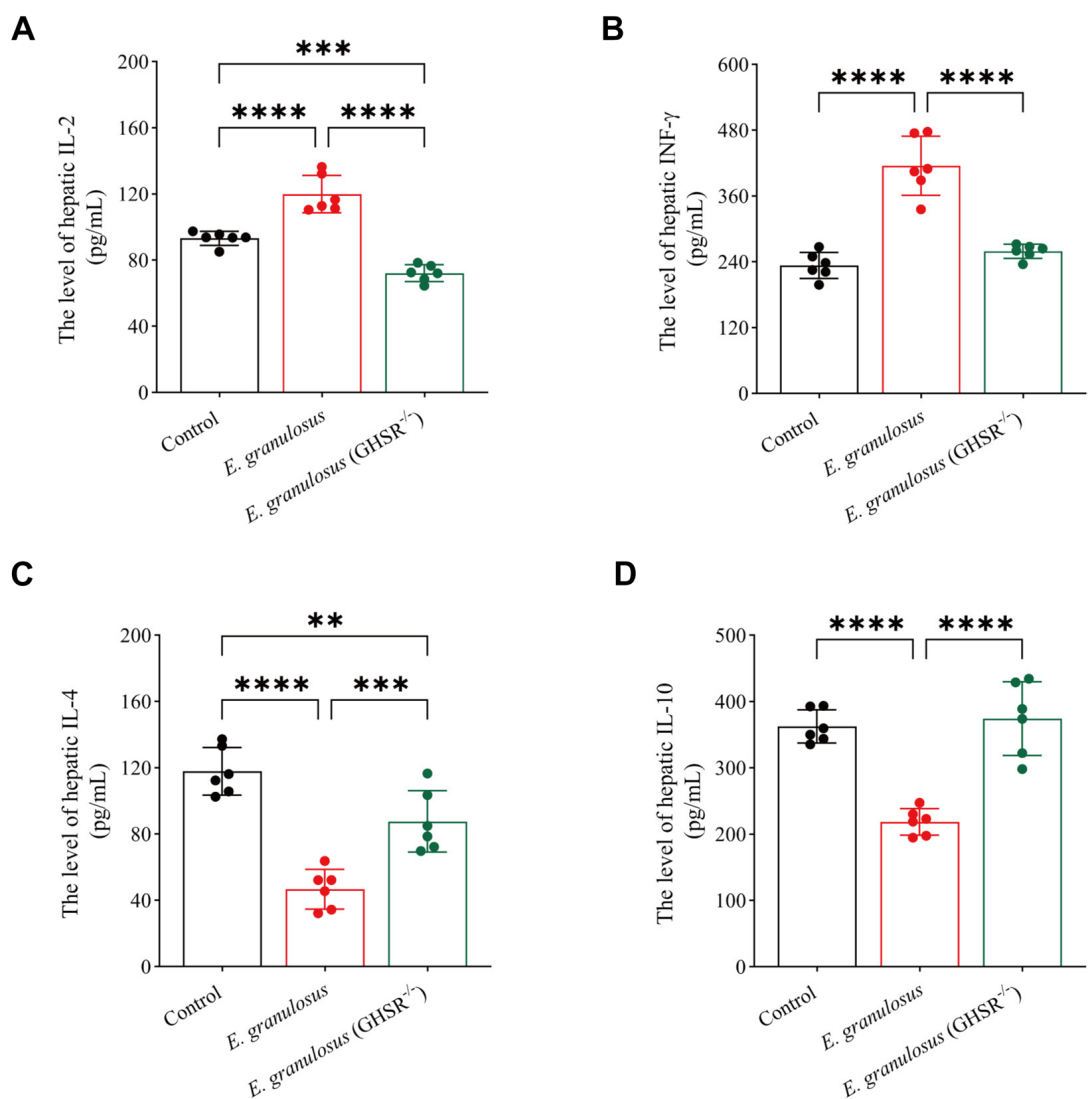
**GHSR deficiency alleviates the hepatic inflammatory response in *E. granulosus* infection. A** ELISA analysis of liver IL-2 levels and comparisons between groups. **B** ELISA analysis of liver IFN-γ levels and comparisons between groups. **C** ELISA analysis of liver IL-4 levels and comparisons between groups. **D** ELISA analysis of liver IL-10 levels and comparisons between groups. Data were considered statistically significant: *P < 0.05; **P < 0.01; ***P < 0.001; ****P < 0.0001.

The expression of proinflammatory cytokines (IL-2 and IFN-γ) and anti-inflammatory cytokines (IL-4 and IL-10) in liver tissue homogenates was measured via ELISA. Compared with the control and GHSR^−/−^ groups, the *E. granulosus* group presented significantly higher levels of IL-2 and IFN-γ (Figures 6A, B) and significantly lower levels of IL-4 and IL-10 (Figures 6C, D). These results suggest that parasitic infection in the *E. granulosus* group disrupted the Th1/Th2 immune balance, promoting a proinflammatory response to clear the infection. In contrast, the GHSR^−/−^ group maintained a Th1/Th2 immune balance similar to that of normal mice, which correlated with a milder infection and reduced immune activation. These findings suggest that immune balance may be preserved in GHSR^−/−^ mice, mitigating excessive inflammation and liver pathology associated with *E. granulosus* infection.

### GHSR deficiency reduces ghrelin and inflammatory factor protein expression in liver lesions and surrounding areas during *E. granulosus* infection

**Figure 7:**
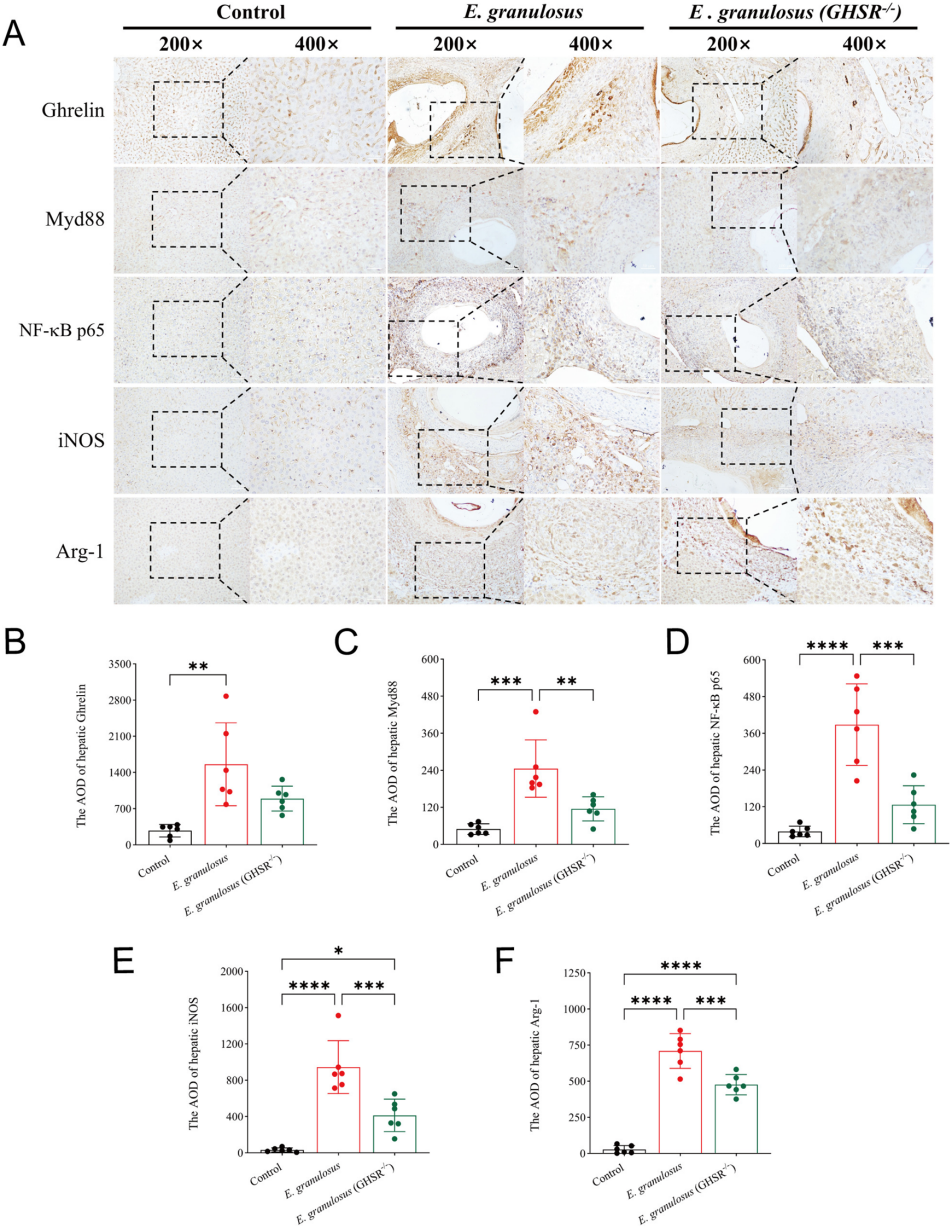
**GHSR deficiency reduces ghrelin and inflammatory factor protein expression in liver lesions and surrounding areas during *E. granulosus* infection. A** IHC staining images of ghrelin and the inflammation-related cytokines Myd88, NF-κB p65, iNOS, and Arg-1 in liver sections. **B** Intergroup analysis of the AOD value of ghrelin protein expression quantified by IHC staining. **C** Intergroup analysis of the AOD value of Myd88 protein expression quantified by IHC staining. **D** Intergroup analysis of the AOD value of NF-κB p65 protein expression quantified by IHC staining. **E** Intergroup analysis of the AOD value of iNOS protein expression quantified by IHC staining. **F** Intergroup analysis of the AOD value of Arg-1 protein expression quantified by IHC staining. Data were considered statistically significant: *P < 0.05; **P < 0.01; ***P < 0.001; ****P < 0.0001.

Immunohistochemistry (IHC) was used to examine the expression of ghrelin and inflammation-related cytokine proteins in the inflammatory cell belt surrounding liver lesions. IHC staining revealed that liver lesions in *E. granulosus*-infected mice presented significantly increased expression of ghrelin, MyD88, NF-κB p65, iNOS, and Arg-1 (Figure 7A). Ghrelin expression was noted in both the peripheral and inner parts of the inflammatory cell belt, while MyD88, NF-κB p65, and iNOS were predominantly expressed in the peripheral regions, and Arg-1 was expressed mainly in the inner regions. These findings suggest that the immune-inflammatory microenvironment around cystic lesions is characterized by an anti-inflammatory response in the inner region and a proinflammatory response in the outer region, with ghrelin being involved in modulating this response. The GHSR^−/−^ group presented a reduction in ghrelin expression in the inflammatory cell belt (Figure 7B), likely due to reduced binding of ghrelin following receptor knockout. Additionally, the expression of MyD88, NF-κB p65, iNOS, and Arg-1 was significantly elevated in the *E. granulosus* group (Figures 7C–F), confirming that GHSR-/- mice exhibited milder infection and a lower inflammatory status, further supporting the conclusion that GHSR deficiency alleviates *E. granulosus* infection.

## Discussion

In this study, we explored the role of ghrelin and its receptor, GHSR, in the progression of *E. granulosus* infection in the liver. The regulatory effects of ghrelin on cytokines and growth-metabolism pathways have been shown to alleviate immune inflammation, fibrosis, and liver damage, potentially mediating the progression of liver CE [7]. In general, ghrelin can exert protective effects by inhibiting immune-inflammatory and fibrotic signalling pathways in liver fibrosis and promoting hepatocyte proliferation and repair, thereby improving disease progression [30–32]. However, during the progression of liver CE, this protective effect may actually facilitate the parasitism and survival of the parasite within the host, accelerating disease progression [33].

Our previous research suggested that blocking the ghrelin receptor, GHSR, could promote the death of *E. granulosus* protoscoleces and potentially slow the progression of liver infection [15]. To validate this conclusion, we used GHSR-KO mice in a liver *E. granulosus* infection model and observed that GHSR-KO significantly improved the progression of liver *E. granulosus* infection. There was a notable reduction in both active cystic lesions and possibly inactive fibrotic lesions within the liver, which is consistent with our previous hypothesis.

Previous studies have indicated that Th1/Th2 immune imbalance is a key factor in immune tolerance and immune evasion by liver cystic echinococcosis parasites. Early *E. granulosus* infection primarily involves a Th1-type immune response to combat parasitic infection [34, 35]. Maintaining a strong Th1-type immune response can provide protective effects, reducing organ damage caused by the parasite. On the other hand, a robust Th2-type immune response promotes immune tolerance and parasite immune evasion, worsening disease progression [25, 26, 36, 37]. In this study, we also revealed that *E. granulosus* infection significantly increased the secretion of proinflammatory cytokines (IL-2 and IFN-γ) in the liver while suppressing the secretion of anti-inflammatory cytokines (IL-4 and IL-10), thereby activating the inflammatory response in the liver to protect the host. In contrast, the GHSR-KO *E. granulosus*-infected mice presented significantly reduced liver inflammation, indirectly indicating that the absence of the GHSR gene improved the progression of liver *E. granulosus* infection, with liver inflammation levels closer to those of normal mice.

We also examined the impact of the GHSR receptor on ghrelin levels during liver *E. granulosus* infection. The ELISA results revealed that *E. granulosus* infection significantly elevated the serum and liver ghrelin levels, whereas GHSR-KO significantly suppressed this elevation. This result indirectly suggests that GHSR-KO may improve the progression of liver *E. granulosus* infection by lowering the activity of ghrelin, which affects the host’s innate immunity and the parasite’s growth and metabolism, thereby improving disease progression. Further IHC analysis supported the role of ghrelin in regulating immune inflammation during liver *E. granulosus* infection. Previous studies have indicated that innate immune pathways, such as inflammatory vesicles and Toll-like receptor activation, as well as hepatocyte apoptosis, are critical defenses against the progression of liver CE infection [38, 39]. In liver cystic lesions and the surrounding inflammatory cell belts of *E. granulosus*-infected livers, we observed high protein expression levels of ghrelin, MyD88, NF-κB p65, iNOS, and Arg-1. Interestingly, ghrelin was expressed in both the peripheral and inner parts of the inflammatory cell belt, while MyD88, NF-κB p65, and iNOS were expressed mainly in the peripheral region, and Arg-1 was expressed mainly in the inner region. This distribution pattern suggests that the immune-inflammatory microenvironment surrounding liver cystic lesions may have a predominant anti-inflammatory response in the inner region and a proinflammatory response in the peripheral region, with ghrelin playing a role in regulating this process. This unique immune‒inflammatory microenvironment likely provides *E. granulosus* with an opportunity for immune evasion, promoting its parasitism and survival within the host.

Notably, in GHSR-KO mice, we observed a significant reduction in the expression of ghrelin and other inflammation-related cytokine proteins in the inflammatory cell belt around the liver lesions. This finding suggests a lower inflammatory status and indirectly reflects that GHSR-KO not only improves the progression of liver *E. granulosus* infection but also reduces ghrelin expression. These data suggest a correlation between ghrelin levels and the development of liver *E. granulosus* infection.

In conclusion, ghrelin and its receptor, GHSR, play important roles in liver *E. granulosus* infection. GHSR gene knockout may improve the progression of liver *E. granulosus* infection and the liver inflammation induced by infection. Furthermore, the GHSR-KO mouse model provides a valuable tool for exploring the specific mechanisms by which ghrelin influences liver *E. granulosus* infection and offers potential therapeutic targets for the development of new treatment strategies. These findings not only enhance our understanding of the pathophysiology of *E. granulosus* infection but also provide new perspectives for future therapeutic interventions.

## Data Availability

All the data generated or analysed during this study are included in this published article.
